# Involving Communities in the Targeting of Cash Transfer Programs for Vulnerable Children: Opportunities and Challenges^☆^

**DOI:** 10.1016/j.worlddev.2013.09.002

**Published:** 2014-02

**Authors:** Laura Robertson, Phyllis Mushati, Morten Skovdal, Jeffrey W. Eaton, Jeremiah C. Makoni, Tom Crea, Gideon Mavise, Lovemore Dumba, Christina Schumacher, Lorraine Sherr, Constance Nyamukapa, Simon Gregson

**Affiliations:** Imperial College London, UK; Biomedical Research & Training Institute, Harare, Zimbabwe; University of Bergen, Norway; Imperial College London, UK; DOMCCP, Manicaland, Zimbabwe; Boston College, Chestnut Hill, USA; Catholic Relief Services, Harare, Zimbabwe; Johns Hopkins University School of Medicine, Baltimore, USA; University College London, UK; Imperial College London, UK; Biomedical Research & Training Institute, Harare, Zimbabwe

**Keywords:** sub-Saharan Africa, Zimbabwe, children, cash transfers, HIV/AIDS, social welfare

## Abstract

We used baseline data, collected in July–September 2009, from a randomized controlled trial of a cash transfer program for vulnerable children in eastern Zimbabwe to investigate the effectiveness, coverage, and efficiency of census- and community-based targeting methods for reaching vulnerable children. Focus group discussions and in-depth interviews with beneficiaries and other stakeholders were used to explore community perspectives on targeting. Community members reported that their participation improved ownership and reduced conflict and jealousy. However, all the methods failed to target a large proportion of vulnerable children and there was poor agreement between the community- and census-based methods.

## 1 Introduction

There is a growing policy emphasis in the field of international public health and development on the need for community involvement in health and development programs (Campbell, Nair, Maimane, & Gibbs, 2009; Wouters, Van Damme, Van Loon, van Rensburg, & Meulemans, 2009). Reflecting the community asset framework (Moser, 1998), the World Bank argues that, through the involvement of community members, a variety of local skills and abilities can be drawn upon in the implementation of social development programs, which, in turn, has the potential to improve local ownership of programs and increase their sustainability (The World Bank, 2011). Involving community members in the identification of beneficiaries of a cash transfer (CT) program may therefore, through its recognition and use of local resources and knowledge, facilitate a sense of local program ownership, in a way survey based targeting tools may not.

### (a) Targeting social welfare programs: census and community participatory approaches

Household censuses are frequently used to collect information for targeting social welfare programs (Ministry of Community and Social Services (MCDSS) & German Technology Cooperation (GTZ), 2007; Robertson *et al*., 2013; Schubert & Huijbregts, 2006). The most vulnerable and/or poorest households can be identified by asking questions about socio-demographic characteristics of households (e.g., orphan status of children in the household, chronic illness among household members, child-headed households, *etc*.) or about household wealth.

Collection of data on household assets, in census questionnaires, is a popular method for obtaining information about household wealth and thereby identifying poor households (Howe, Hargreaves, & Huttly, 2008). This method makes use of simple questions and data on several household assets can be used together to create a wealth index by which households can be ranked and the poorest households thus identified (Howe *et al*., 2008). Direct observation of assets by the interviewer can reduce recall and social-desirability bias compared with other methods—e.g., data on household expenditure or income, which often vary significantly over short time periods and for which reporting may be influenced by social norms on the acceptability of discussing household wealth. Studies suggest that the extent to which asset-based wealth indices correlate with other indicators of poverty (e.g., household consumption expenditure data) varies by country (Sahn & Stifel, 2003). A study using data from India, Pakistan, and Nepal found that asset-based wealth indices were associated with school-enrollment and could predict school-enrollment as accurately as household expenditure data (Filmer & Pritchett, 2001).

One advantage of using a population-based census is that it is relatively simple to ensure the systematic application of a standardized questionnaire across an entire population. An important disadvantage is that large-scale censuses are expensive and time-consuming to carry out. Furthermore, there are often few opportunities for community involvement in census-based targeting. If external definitions of vulnerability and poverty are used, communities may feel resentment toward the associated social welfare programs and it could cause conflict within the community.

Alternative targeting methods that directly involve community members in the targeting process are one means of achieving community participation. For example, a group of community representatives could be responsible for identifying vulnerable households (Pronyk *et al*., 2006) or could use census data in making the final decision about which households should be selected (Miller, Tsoka, & Reichert, 2008; Ministry of Community and Social Services (MCDSS) & German Technology Cooperation (GTZ), 2007). Participatory wealth ranking (PWR) is a method for involving communities in the selection of the poorest households (Grandin, 1988; Hargreaves *et al*., 2007). Meetings are held with community representatives to discuss the characteristics of households in different wealth categories (e.g., poorest, average, least poor, *etc*.). The representatives then use these categories and characteristics to rank the households in the community according to their wealth status and thus the poorest households can be identified. Community-based methods allow information about household wealth and vulnerability to be generated relatively quickly and cheaply. Studies from Tanzania (Temu, 2000) and southern Zimbabwe (Scoones, 1995) found participatory wealth ranking data correlated well with wealth indices based on household-level agricultural wealth (e.g., crop sales, livestock ownership, land ownership, *etc*.). However, Hargreaves *et al*. (2007) compared wealth indices based on a wider range of variables (e.g., employment status, household assets, details of dwelling construction, *etc*.) with data generated using participatory wealth ranking and found only limited agreement between the two methods for a population in rural South Africa.

### (b) Targeting cash transfer programs in sub-Saharan Africa

Cash transfer programs are social welfare interventions that aim to help households meet their basic needs and provide care for vulnerable children (Adato & Bassett, 2009). In conditional cash transfer programs, beneficiary households must meet certain conditions, usually relating to school attendance and uptake of health services, in order to receive the transfers. Unconditional cash transfers are provided without conditions.

National cash transfer programs in Latin America (e.g., Progresa in Mexico (Skoufias, Davis, & Behrman, 1999)) use household-level means testing based on routinely collected data on income to target children living in the poorest households. In sub-Saharan Africa, these data are often unavailable. Programs in Zambia (Ministry of Community and Social Services (MCDSS) & German Technology Cooperation (GTZ), 2007) and Malawi (Miller *et al*., 2008) targeted “ultra-poor, labor-constrained households” by identifying households with high ratios of dependents (children, elderly and sick adults) to working-age adults. Demographic and economic data were collected from potentially vulnerable households identified by community committees. These data were then used to rank households based on their level of destitution and community committees discussed and verified the list and identified the 10% most incapacitated households. This method was designed to be simple and to target economically vulnerable households and/or those suffering from the demographic consequences of the HIV epidemic (i.e., the illness and death of working-age adults).

Attempts to rigorously evaluate these targeting methods, in the context of cash transfer programs in sub-Saharan Africa, have been limited. A study in Zambia found that targeted households were more likely to be elderly or single-headed or to contain orphaned children or disabled members (Ministry of Community and Social Services (MCDSS) & German Technology Cooperation (GTZ), 2007). A study from Malawi found that targeted households were more likely to be caring for orphaned children or someone sick with HIV or TB (Miller *et al*., 2008). However, it remains to be established whether census-based or community-based participatory methods perform better with respect to reaching the most vulnerable children.

There are also questions pertaining to the appropriateness and accountability of cash transfers to beneficiaries and their wider community. To date, little has been done to incorporate and bring forward the perspectives of beneficiaries, let alone report on their experiences of engaging with cash transfers. A recent report, reviewing the experiences of beneficiaries and implementing stakeholders of five major unconditional cash transfer programs in sub-Saharan Africa, identified a need to promote community participation in poverty alleviation programs in order to secure greater accountability and program responsiveness to local needs and program shortcomings (Samuels, Jones, & Malachowska, 2013). This paper contributes directly to policy and program recommendations on community participation in the targeting of cash transfers.

### (c) Manicaland cash transfer trial

From 2009 to 2011, we conducted a community-randomized controlled trial of a cash transfer program for orphaned and other vulnerable children (OVC) in Manicaland, eastern Zimbabwe (Robertson *et al*., 2013). The program was funded by the Program of Support for the Zimbabwe National Action Plan for Orphans and Vulnerable Children (UNICEF Zimbabwe). We investigated the effects, on school attendance and the uptake of child vaccinations and the uptake of birth registration, of a conditional cash transfer program and an unconditional cash transfer program. Every two months, beneficiary households received US$18 plus US$4 per child in the household up to a maximum of three children.

We did not use an experimental design to compare different targeting methods. However, we used a combination of survey-based and community participatory methods to target vulnerable households caring for children. This provided us with an opportunity to compare census and community derived targeting information. In the baseline survey for this trial, we collected data on household-level targeting information and child development indicators (i.e., the trial endpoints). As part of the evaluation of the cash transfer program, we also investigated community responses to the program through focus group discussions and key informant interviews with program beneficiaries, those delivering the program, and other community members.

In this paper, we investigate and compare, from several perspectives, the success of community- and census-based targeting methods for cash transfer programs for vulnerable children. To determine whether our census- and community-based targeting methods successfully enumerated all households in the study areas and whether they identified the same households as vulnerable, we compared household eligibility data collected in the baseline census with eligibility data collected through community participatory methods. We then compared the effectiveness, coverage and efficiency of census- and community-based methods in reaching children with poor developmental indicators. Finally, in light of these findings, we use qualitative data to explore community perspectives on the benefits and challenges of involving community members in the selection of cash transfer beneficiaries.

## 2 Methods

### (a) Study region

Manicaland province is located in eastern Zimbabwe, on the border with Mozambique. Many households in the region make their living from agriculture, both subsistence agriculture and in large scale commercial tea and tobacco estates. From 1999, Zimbabwe experienced severe economic decline, with record levels of hyperinflation that peaked around 2008 and then stabilized in 2009. In 1998–2000, HIV prevalence in Manicaland was 25.3% in women and 18.8% in men aged 15–49 years (Schur *et al*., 2013). By 2006–2008, the prevalence had fallen to 18.7% in women and 12.5% in men (Schur *et al*., 2013). Orphan prevalence is high in the region: 20.8% of children aged 0–14 years had lost at least one parent in 2003–2005 (Robertson, 2010).

### (b) The Manicaland Cash Transfer trial targeting process

The Manicaland Cash Transfer trial for OVC began in July 2009 in 30 communities with an average of around 400 households in each community. The communities comprized four socio-economic strata—small towns, roadside settlements, subsistence farming areas and large-scale agricultural estates. The cash transfer programs were designed to support children in households that had been affected by extreme poverty and/or the severe demographic impacts of the HIV epidemic. An initial feasibility study was conducted to identify important indicators of household vulnerability, including a vulnerability mapping exercise based on national data on vulnerable children and discussions with community members and other stakeholders (Robertson *et al*., 2013). It was decided to target all children within vulnerable households to avoid conflicts that could arise if specific children within households were singled out for assistance. Only households caring for children were eligible for the program.

Households were eligible for the cash transfer program if they cared for at least one child aged less than 18 years, were not in the richest 20% of households and met at least one of the following criteria: was in the poorest 20% of all households (defined below), cared for orphans (<18 years), had a household member with a chronic illness or disability, or was a child (<18 years) headed household. Data on household eligibility were collected in a baseline household census. Lists of all households in the communities were compiled from lists of households that had ever been enumerated in an on-going cohort study in the area. This cohort study had performed a census in the area every two or three years since 1998 (Gregson *et al*., 2006). New households were added to the list as they were encountered during the survey. Local guides from each community asked representatives from the households in their area to convene at a central meeting point on a specific day. Each central meeting point was visited on three different days. Trained research assistants conducted interviews, in the local language *Shona*, with the most senior available member of each household.

To identify the poorest 20% of households, data were collected on household assets—source of drinking water, type of toilet facility, type of house, type of floor in the main dwelling, ownership of a radio, a television, a motorbike or a car and whether or not the household had its own electricity. The household asset data were used to create a wealth index for all households in the study using a simple summed score of asset ownership. The households were then ranked for each community, based on this index and the poorest 20% were identified. The summed score index was developed and validated using data collected previously in Manicaland (Lopman *et al*., 2007).

Data on household socio-demographic eligibility criteria (chronic illness or disability among household members, age of the household head, and parental survival status of all children <18 years) were also collected. We used the following definition of chronic illness: very sick for at least 3 months during the past 12 months, where “very sick” was defined as being too sick to work or do normal activities around the house. We also asked whether any household members had any form of disability. Children were defined as orphans if either of their parents were deceased.

Following the census, lists of all households in the study clusters, along with their status with respect to the various eligibility criteria were prepared and passed to a local NGO who undertook a community-based targeting process. Small groups of community leaders, including village chiefs, village heads, councillors and other representatives, nominated by the community during sensitization meetings (where the project and its aims were explained to the local communities) performed a participatory wealth ranking (PWR) procedure. The groups, led by the local NGO, were asked to define characteristics of “poorest”, “poor”, “average”, “less poor” and “least poor” households. Using these characteristics as a guide, the groups were then asked to rank the households on the census lists by assigning each household to one of the five categories listed above. Equal numbers of households were intended to be assigned to each category so that the poorest 20% could be identified. Larger community meetings were also held to verify the accuracy of the household socio-demographic eligibility data. The members of these groups were familiar with the households in their area. During the trial, eligible households were identified using the survey and the community-based participatory methods. A household had to be identified as eligible by both the census- and community-based targeting methods in order to be enrolled in the cash transfer program.

### (c) Quantitative methods

We compare the distribution of household wealth and socio-demographic vulnerability characteristics between the census data and the community information among households caring for at least one child less than 18 years. We assess agreement between community- and census-based information about binary socio-demographic variables using simple kappa statistics (Kirkwood & Sterne, 2003). For the wealth quintile data, which are ordered categorical variables, we use a weighted kappa statistic, which accounts for poorer agreement between two measures if the measurements disagree by more categories (e.g., if one measurement finds a household to be in the poorest category and another measurement finds them to be in the least poor category; that is worse than if the measurements find the household to be in the poorest and the poor categories, respectively).^1^ Complete agreement between two measurement methods produces a kappa statistic of one. If there is no more agreement than would be expected by chance then the kappa statistic will be zero. We used Landis and Koch’s criteria for assessing kappa statistics: values greater than 0.75 represent excellent agreement, values between 0.4 and 0.75 represent fair to good agreement and those below 0.4 represent moderate or poor agreement (Landis & Koch, 1977).

For the ordered categorical wealth data, the maximum value of the weighted kappa statistic is affected by the distribution of households across different wealth categories—the asset-based wealth index and the PWR procedure may rank households in roughly the same order but if the communities did not assign equal numbers of households to each category during the PWR (as instructed), then the kappa statistic would be reduced when comparing this distribution with quintiles calculated using the wealth index. As a comparison to the weighted kappa statistic based on comparing the asset-based wealth quintiles with the wealth distribution produced by the PWR procedure, we calculated the maximum possible weighted kappa statistic that could be produced by this method if the households were ranked in the same order by the two procedures, but differed with respect to the size of the wealth categories as observed. Secondly, we calculated a weighted kappa statistic comparing the wealth categories produced by the PWR procedure with those produced when the wealth index ranked household list was divided into categories with the same distribution as those produced by the PWR procedure (instead of strict quintiles).

In the baseline census, data were also collected on the primary outcome indicators for the trial: birth registration and vaccination status (polio, BCG, measles, and DPT) among children aged 0–4 years and school attendance among children aged 6–17 years. These primary indicators were selected to represent various types of health, education, and social vulnerability among children across a range of ages. We defined four poor child-level outcomes: incomplete vaccination record among children 0–4 years, lack of a birth certificate among children aged 0–4 years, non-enrollment in school or less than 80% attendance over the last 20 school days (i.e., poor school attendance) among children aged 6–12 years and children aged 13–17 years.

We used these data to compare the effectiveness and efficiency—with respect to reaching children with poor health, education, and social outcomes—of targeting the poorest households identified by the PWR procedure and the poorest 20% of households based on the asset-based wealth index. To account for differences in the proportions of children assigned to the “poorest” category by the PWR procedure and the asset-based wealth index quintiles, we also defined a targeting method based on the asset-based wealth index ranking that identified the same proportion of “poorest” children as the PWR procedure (instead of strictly the poorest 20%). We also defined two targeting methods that combined the PWR procedure and the asset-based index: an “inclusive” method where any child considered to be in the “poorest” category based on either the PWR procedure or the asset-based wealth quintiles would be targeted and an “exclusive” method where a child would need to be considered to be in the “poorest” category by both methods to be targeted.

The effectiveness of each of the five poverty-based targeting methods at reaching children with poor outcomes was compared using age- and sex-adjusted logistic regression models to estimate the odds-ratio that targeted households contained children with poor outcomes relative to households not targeted by each method. To compare the extent to which children with poor outcomes were “missed” by each method, we present the proportion of children with poor outcomes that were reached and compare this with the proportion of all children that are reached by each method. We compared the efficiency of the methods by calculating the number of children with each of the poor outcomes that were reached per child targeted.

Socio-demographic information on child headed households and the orphan status, chronic illness status, and disability status of household members were not collected independently in the household census and the community-based participatory data collection: the community groups verified the data collected in the census rather than generating their own data (they generated their own wealth data independently of the household census when they performed the PWR procedure). We therefore have not compared the effectiveness of census-based and community-based socio-demographic targeting of vulnerable children. Comparisons between socio-demographic and poverty-based targeting methods have been presented elsewhere (Robertson *et al*., 2012).

### (d) Qualitative methods

To explore community perceptions of the cash transfer program, including the procedures used to identify and select eligible households, we conducted 35 individual interviews and 3 focus group discussions. In an effort to gather a wide range of perspectives, we invited community members with different types of involvement in the cash transfer program, including: 7 (of which one was an adolescent, age 14) cash transfer beneficiaries; 8 (of which 3 were youths between the ages 15 and 21) conditional cash transfer beneficiaries; 5 non-beneficiaries; and 15 key informants who contributed to the implementation of the program within the communities (see Table 1). The participants were randomly selected from a list of program stakeholders and recruited by Shona-speaking researchers from the Biomedical Research and Training Institute in consultation with local community guides. The qualitative work was not conducted at the same time as the community-based participatory targeting procedures.

With the exception of one individual interview, which was conducted in English, all interviews were conducted in the local Shona language, using a topic guide developed specifically to explore their perspectives on the cash transfer program. The interview guides covered topics such as the role of community members in the implementation of the program, procedures and performance of targeting methods, changes to community life as a result of the cash transfers program, the impact of cash transfers on the benefitting households, compliance, and monitoring procedures and challenges as well as recommendations for future programs. The individual interviews lasted an average of 40 min, while the group interviews took an average of 94 min. The interviews were translated and transcribed into English and imported into Atlas.ti v6.1 (ATLAS.ti Scientific Software Development GmbH, Berlin), a qualitative software package, for coding and examination. This involved an iterative process allowing for both a priori reasoning and surprises. This first stage of the analysis generated a total of 90 codes. In line with Attride-Stirling’s thematic network analysis (Attride-Stirling, 2001), codes were clustered together into more interpretative organizing themes. As we did not seek to report on all the themes emerging from our qualitative analysis in this paper, but to examine community perspectives on the interface between their involvement and support of the program, we report on three organizing themes, which comprise of 19 codes, or basic themes, that have direct relevance to this topic and contextualize our quantitative findings. Table 2 illustrates the breadth of related themes emerging from this study, giving detail to: how the program worked within local structures; the community committees active involvement in implementation; perceived benefits of participatory wealth ranking; community verification; perceptions of fair selection; transparency; the limits of community involvement.

## 3 Results

We first present our quantitative findings, comparing the households identified by the census-based and community-based targeting methods and investigating the relative effectiveness, coverage, and efficiency of these targeting methods. We then supplement and contextualize these findings with community members’ perspectives of the different targeting methods, highlighting additional benefits and challenges of involving community members in the selection of cash transfer beneficiaries.

### (a) Census-based targeting methods and community-based participatory targeting methods: do they target the same households?

A total of 16,887 households were identified as having been enumerated in at least one census since 1998 of which 11,820 households (70%) completed a household census as part of the cash transfer study. Of those who did not complete a census, only 10 (0.06%) refused to be interviewed. The rest had either relocated (2,358, 14%) or their dwelling was empty or no longer existed (1,836, 11%). For 863 missing households (5%), the reason they were not interviewed was unknown. Of those households interviewed, 10,538 (89%) cared for at least one child under 18 years old.

The coverage of the community-based participatory wealth ranking (PWR) and of the socio-demographic verification was less complete than the coverage of the household census—2,455 (23%) of households caring for children that completed a census were missing PWR data and 899 (9%) were missing community verification of socio-demographic characteristics. Using the census data, we compared households missing data with households that were not missing data (Table 3). Households missing PWR data were significantly less likely to have poor socio-demographic vulnerability characteristics. Few significant differences were found between households missing community-based socio-demographic verification data and households not missing these data: households missing data were less likely to be female-headed and children 6–12 years living in households with missing data were more likely to have poor school attendance.

**Figure 1:**
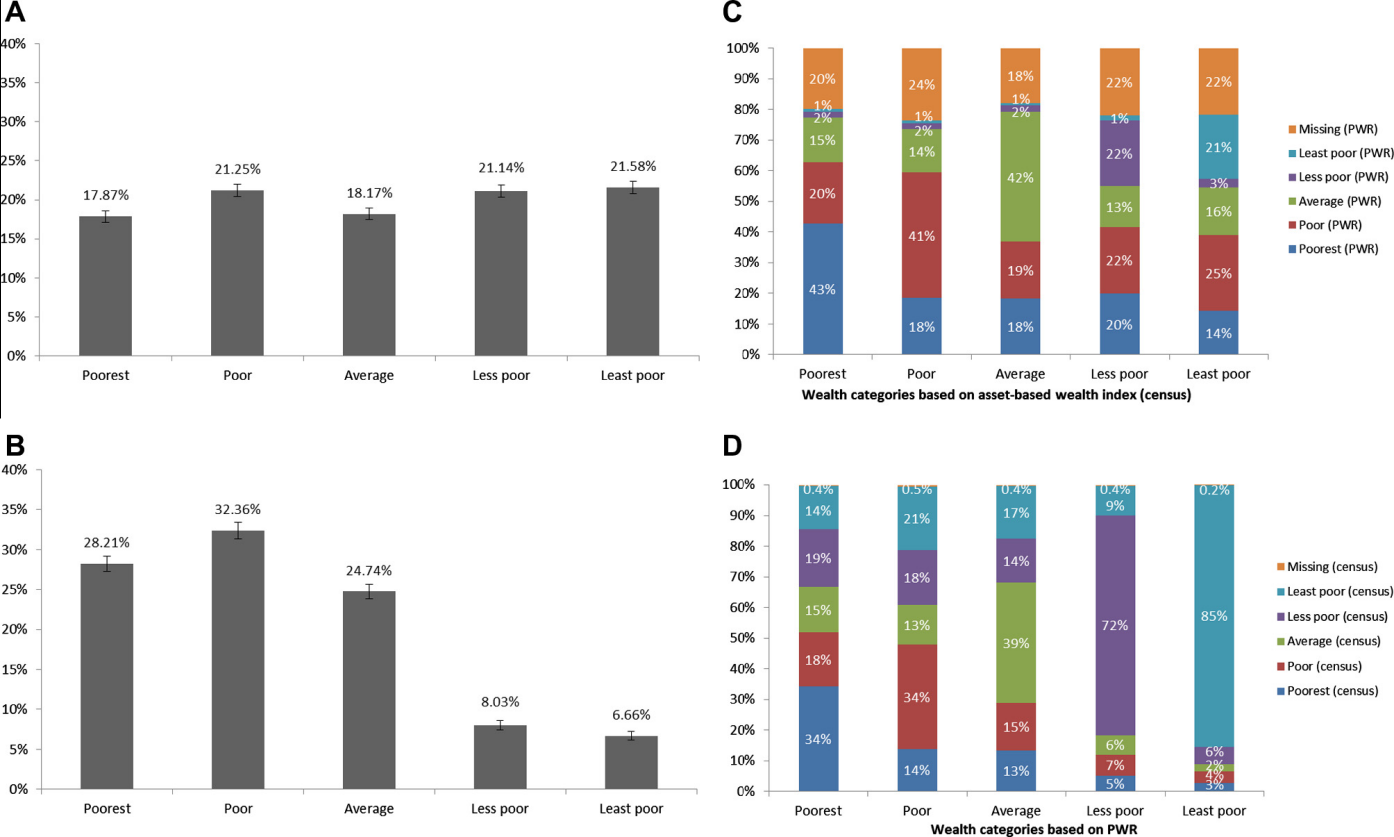
Distribution of household-level wealth in Manicaland based on an asset-based wealth index and a participatory wealth ranking procedure (PWR)—(A) Wealth distribution of households caring for children less than 18 years (asset-based wealth index); *N* = 10,484; (B) Wealth distribution of households caring for children less than 18 years (PWR); *N* = 9,262); (C) Wealth distribution based on the asset-based wealth index broken down by the PWR wealth distribution; and (D) Wealth distribution based on PWR broken down by the asset-based wealth index (census) distribution.

Panels A and B of Figure 1 show the distributions of household wealth using information on household assets from the population census and information from the PWR procedure. Using the asset-based wealth index, we divided the population roughly into equal sized wealth quintiles. The slight variation in the size of the categories, including 18% of households being in the poorest category, is due to the fact that many households had exactly equal scores in the wealth index, which resulted in category cut-off points slightly above or below the quintile cut-off points. Using the categories produced during the PWR procedure, it is clear that households were not evenly distributed across the five wealth categories by the community groups: 28% were assigned to the poorest wealth category and very few households were assigned to the two least poor categories. Instructions to the community groups to assign households evenly across the five wealth categories were not well followed.

A poor level of agreement was found between the PWR categories of household wealth and the asset-based index quintiles—the weighted kappa statistic was 0.28, although the maximum possible weighted kappa value, assuming both procedures ranked households in the same order but differed with respect to the sizes of the wealth categories, was 0.54. When we compared the level of agreement between the PWR categorization and the asset-based wealth index categories produced to match the size of the PWR categories, the level of agreement remained low (weighted kappa = 0.18).

Panel C of Figure 1 shows the breakdown of the households into wealth categories based on the PWR procedure for each quintile of the asset-based wealth index. Households in the poorest quintile of the asset-based wealth index had the highest proportion of households assigned to the poorest category by the PWR procedure. A somewhat similar pattern was found across all quintiles—a large proportion of households in each asset-based wealth index quintile were assigned to the analogous PWR category. However, across all the wealth quintiles, a substantial proportion (around 40%) of households were assigned to the poor or poorest categories by the PWR procedure, including among households in the less poor and least poor asset-based wealth index quintiles.

In panel D of Figure 1, the distribution of households according to the asset-based wealth index is shown for each wealth category from the PWR procedure. Those households categorized in the poor or poorest categories by the PWR procedure were much more likely to be in the poorest two quintiles of the asset-based wealth index. Among the households categorized as better-off by the PWR procedure, there were high proportions in the richer quintiles of the wealth index. Very few households categorized as less poor or least poor by the PWR were in the poor or poorest quintiles of the wealth index, although a relatively large proportion of households categorized as poorest or poor by the PWR were in the better-off two quintiles of the wealth index.

Table 4 shows the proportion of households with each of the socio-demographic characteristics of vulnerability according to the household census and the community verification exercise and the kappa statistics measuring the agreement between these two information sources. For most characteristics, there is good agreement between the census data and the community verification exercise, with the strongest agreement found for identification of paternal orphans in the household. A low kappa statistic was found for agreement in the identification of child-headed households. Child-headed households were found to be very rare in the census data and the community verification data.

Among households with non-matching data socio-demographic data, census based information was significantly more likely to indicate a chronically-ill resident (63.1% *vs*. 36.9%; *N* = 2,193; *p* < 0.001 [*T*-test with null hypothesis of a 50:50 split between census and community data among non-matching households]), a disabled resident (67.6% *vs*. 32.4%; *N* = 750; *p* < 0.001), or a paternal (55.6% *vs*. 44.4%; *N* = 1,679; *p* < 0.001) or double (53.9% *vs*. 46.1%; *N* = 1,197; *p* = 0.007) orphaned household member than the community verification exercise. There were no significant differences, among households with non-matching data, between the census- and community-based information for reporting of maternal orphans (47.8% *vs*. 52.2%; *N* = 1,505; *p* = 0.084) or child-headed households (46.5% *vs*. 53.5%; *N* = 99; *p* = 0.485).

### (b) Comparing the effectiveness, coverage and efficiency, with respect to reaching children with poor outcomes, of the asset-based wealth index and the PWR targeting methods

Children living in households targeted by the PWR procedure (28% of households were categorized as “poorest”), the asset-based wealth index (targeting the poorest 18% or the poorest 28%), and the combined targeting methods, both inclusive and exclusive, were significantly more likely to have poor school attendance and to lack a birth certificate than non-targeted children (Table 5; Section 1). These associations were stronger for the asset-based wealth index methods than for the PWR procedure. The strongest associations were found for the asset-based wealth index targeting the poorest 28% of households. When the two targeting methods were combined inclusively and exclusively, the strengths of the associations were midway between the strengths of the asset-based wealth index associations and the PWR procedure associations.

**Figure 2:**
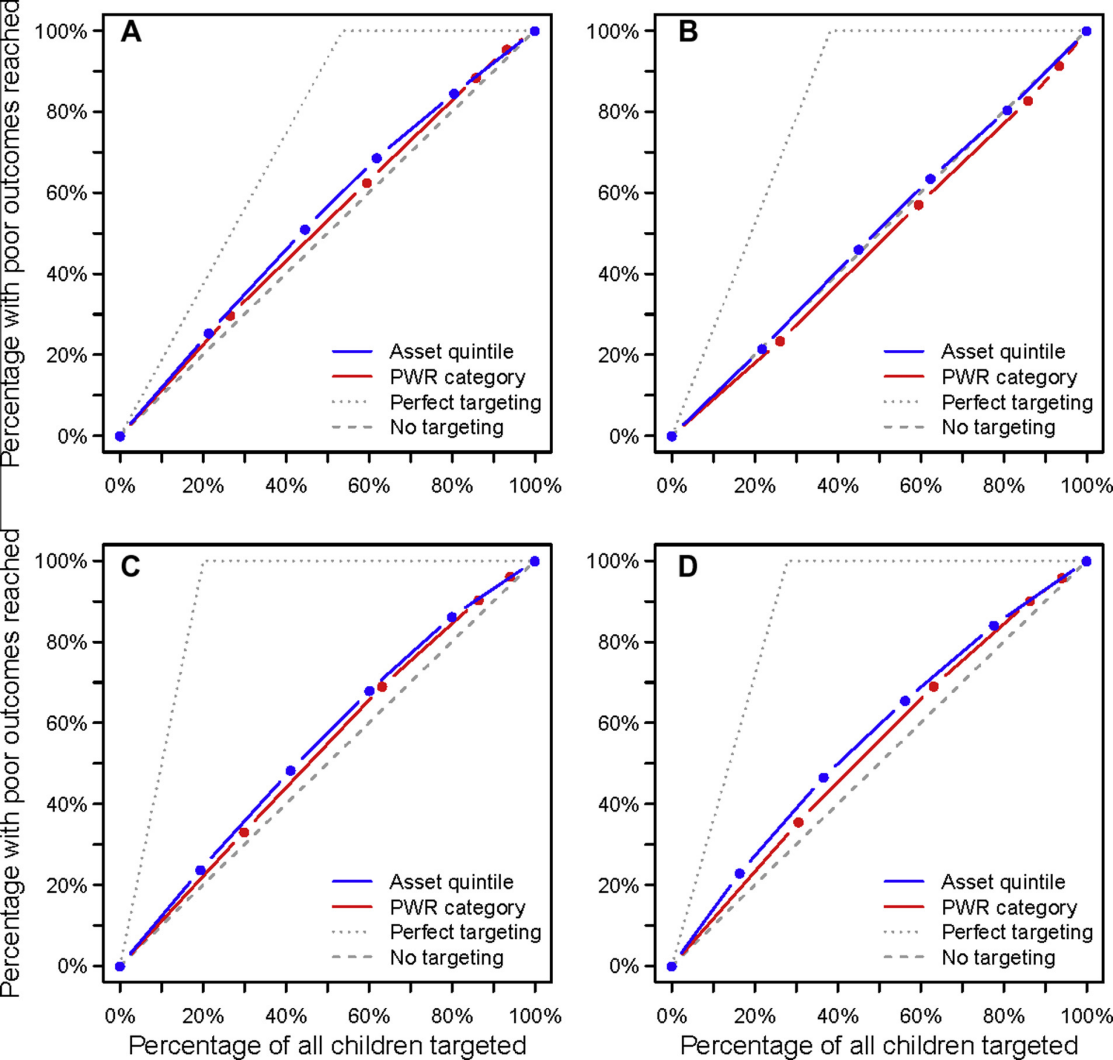
Comparing the proportions of children with each poor outcome reached, by the asset-based wealth index and the PWR procedure, with the proportions of all children reached by each method. (A) Birth not registered among children 0–4 years, (B) incomplete vaccination record among children aged 0–4 years, (C) poor school attendance among children aged 6–12 years, and (D) poor school attendance among children aged 13–17 years. “Perfect” targeting assumes that only children with poor outcomes are targeted. No targeting assumes children are selected at random. Dots on the lines indicate the wealth quintiles or categories for the asset-based wealth index and PWR procedure, respectively.

In Table 5 and Figure 2, we compare the proportion of children with each poor outcome that are reached by each of the targeting methods with the proportion of children in the general population that are reached. Table 5, Section 2 shows that a large proportion of children with poor health, education, and social outcomes are missed by all five targeting methods (all methods reach less than 50% of children with poor outcomes). Figure 2 compares the efficiency of asset-based wealth index and the PWR method at targeting children with each poor outcome by showing the percentage of children with poor outcomes who are reached as the percentage of all children targeted are increased. For birth registration and primary and secondary school attendance, both methods perform slightly better than by chance in reaching children with poor outcomes. The asset-based wealth index performs slightly better than the PWR method for all three of these outcomes. Neither method performs better than chance with respect to reaching children with incomplete vaccination records.

Table 5, Section 3 shows that the efficiency of all five targeting methods was poor—the number of children with poor outcomes reached per child targeted was low for each of the four child vulnerability indicators. All methods were most efficient at targeting children aged 0–4 years who lacked a birth certificate. The asset-based wealth index methods and the exclusive combination method were slightly more efficient than the PWR procedure and the inclusive combination method for all indicators. As the proportion of children reached by the asset-based wealth index increased from 18% to 28%, the efficiency of the method did not change significantly.

### (b) Additional benefits and challenges of involving community members in the selection of cash transfer beneficiaries: a community perspective

A key theme that emerged from our qualitative interviews was the added value of community involvement—manifested through the PWR procedure and community verification process—in facilitating program ownership. One community leader, when discussing pathways to impact, attributed the sense of the success of the program to “the local way of doing things”:

“The programme was successful because it valued people’s input… it drew from the local way of doing things. Above everything I also saw that you know that local leaders are important in your activities and you always take your time to explain your projects to them.” Community leader

The links between program success and the program’s recognition of local structures and “way of doing things” were articulated in many different ways. For example, there was a sense that the PWR procedure gave the community members an opportunity to consider a whole variety of locally relevant information that they believed determined the vulnerability of children. For example, in answering the question: “How did local people define eligible households?” one committee member said:

“People were looking at things like whether the household lives a better life and whether any family member is gainfully employed and bringing in meaningful income. You see there are people who can’t even afford fees for their children. So we were also looking at whether the household has any orphans they were taking care of, and also whether they were struggling to make ends meet. Of course, the issue of ownership of livestock was looked at but generally many people do not have many cows, even some well-to-do families here might not even own any livestock, but people know each other’s living standards. There are families here whose members are handicapped such that they can’t do the daily duties like many of us here. Just by mentioning the name people will tell you whether that household is deserving or undeserving.” Committee member

But it was not just the fact that the PWR procedure allowed for local information to guide the selection of beneficiaries that made it a favorable targeting method. In a response to a question on the benefits of having community meetings to rank vulnerable households, one community member said that the PWR procedure encouraged widespread community involvement, which, in turn, contributed to transparency and the identification of the most vulnerable children.

“The advantage of that process [PWR] is that everyone will be present at the meeting and they will be hearing the selection process. They will know how the households have been ranked and confirm that the household deserve to be in the category they have been placed.” Community member

Both of these observations are supported by a community leader, who further adds that community members know each other, and their situations, better than any outsider and are thus in an ideal position to identify the most vulnerable children. He also adds that community involvement fosters ownership and an interest to see the objectives of the program being met.

“I also think people know each other better than anyone from outside... they actually know who should benefit first. This kind of selecting avoids the possibility of undeserving households benefiting. There is also another advantage whereby the community will assume the responsibility of making sure the program is successful because they won’t have anyone except themselves to blame if the program fails to achieve desired goals.” Community leader

Another point raised by a program implementer, was how the involvement of community members contributed to a sense of transparency and fairness, reducing community conflict and the probability of anyone feeling jealous:

“We know each other better than any outsider, so I felt having the PWR was really a good way of involving the community and making sure there is transparency because at the end of the day this was money and everyone wanted it, but when the most deserving get it no one would cry foul, or accuse any from the implementing agencies to have done anything wrong. I think if they had just sat somewhere and came up with names of beneficiaries, people would have complained, but now in this case no one can complain as it was for the community to decide who should get preference.” Program implementer

Not only did community involvement contribute to program buy-in and acceptance from the community at large, beneficiaries also spoke about the changes they had witnessed in their community, typically referring to it as “united” or “strengthened”:

“This program brought unity to people in our community. The community has been strengthened.” Adult CT beneficiary

These local observations suggest that involving community members in the targeting process encouraged them to have a stake in the program—working toward its success—presenting additional benefits to the PWC procedure and community verification process.

Nonetheless, while community involvement was generally seen as key to the success of the program, some community members felt there should be a limit to what responsibilities should be passed onto them, arguing that they should not be doing the job of the implementing NGO. While they saw community involvement as positive and a prerequisite, they felt the responsibility should not fall on them—highlighting an important challenge in finding a balance between top-down and bottom-up program implementation that is acceptable for everyone.

“We don’t want the involvement of the local people. We want you to do your job. We don’t want the responsibility to fall on local people. We want you working with community members and working with us as one team.” Community member

Furthermore, community participation is notoriously challenging. Any community is characterized by people with competing interests and power relations. Favoritism, nepotism, and lying were mentioned as a challenge to community involvement. One respondent said that “there are some people who ask: “why was this person selected and not me? It must be down to favoritism and nepotism.” Although it is difficult to ascertain whether such a claim is down to jealousy or real observations, it represents a concern. In discussions on the challenges of community involvement, it emerged that some community members had tried to manipulate the eligibility criteria for the benefit of themselves or others, but that the transparent process of involving the community had minimized such attempts.

“There are not many non-deserving children who got into the program. Some people tried to cheat by stating that they were orphans and include their children as orphans. They will end up as 4 people. But these problems were resolved […] by asking the selected households to bring their children’s birth certificates and parent’s death certificates to verify the orphan status of the child.” Community member

Overall, the qualitative data highlighted the importance of involving community members in targeting cash transfer programs in order to capitalize on existing local resources, enhance ownership of the program, improve transparency and perceptions of fairness, and reduce potential for jealousy and conflict.

## 4 Discussion

The benefits of community involvement in the targeting of cash transfer programs include enhanced community ownership, increased transparency, and reduced potential for conflict and jealousy. Making use of existing local resources also reduced the need to build parallel structures. However, our quantitative analysis showed that there was poor agreement between the community-based wealth ranking procedure and an asset-based wealth index in terms of describing the distribution of wealth in Manicaland and identifying the poorest households. A similar result was found when comparing PWR data with survey data collected in rural South Africa (Hargreaves, Morison, Gear, Kim, *et al*., 2007). We did not find that the poor agreement was attributable to the communities’ failure to assign equal numbers of households to each of the wealth categories, as was intended.

Community groups undertaking PWR procedures may take into consideration factors that are not directly related to household wealth (e.g., whether a household member is sick) when ranking households. This could explain to some extent the poor agreement between the PWR ranking and the asset-based index. However, we did not collect quantitative or qualitative data, other than the assignment of households to the five poverty categories, during our PWR procedure. Thus we are unable to investigate further the information that was used by communities to rank the households. In the South African study (Hargreaves, Morison, Gear, Kim, *et al*., 2007), community groups did discuss non-wealth related characteristics, such as the presence of orphaned children in households, when undertaking the PWR. Investigating possible reasons for the lack of agreement between PWR and asset-based wealth indices is an important area for future work.

It is not clear if the reason for the disproportionate assignment of households to the five wealth categories was because the community representatives were inadequately trained in the methodology or whether they found the method unacceptable. It may be the case that they adapted the PWR procedure in accordance with the perceived needs of the community. For example, the PWR-based wealth distribution may more accurately represent the distribution of wealth within the population than wealth quintiles—it is likely that many households in the area are extremely poor and few are extremely rich. The community groups were instructed to discuss characteristics of households in different wealth categories (“poorest”, “poor”, “average”, “less poor”, and “least poor” households) and then use these characteristics to rank the households and assign equal numbers of households to each of the five wealth categories. If large numbers of households had characteristics that the communities associated with the poorer households, this may explain the observed wealth distribution produced by the PWR procedure, with households in the richer wealth index quintiles being categorized in the poorer PWR categories.

Many households were missing PWR and socio-demographic verification data from the community. It is not clear exactly why this happened. There was some evidence that those households that were excluded from the PWR procedure were significantly less likely to have vulnerable socio-demographic characteristics or to be caring for children with poor outcomes. This suggests that the community groups were excluding some better-off households from the ranking procedure. This may have been because such households were less well-known within the communities—perhaps because they were less involved in community activities. It is also possible that the community groups preferred not to consider ranking households they perceived were not in need of assistance. Households excluded from the PWR procedure were more likely to be caring for children with poor school attendance. In this population, recent migration has been found to be associated with school drop-out among vulnerable children (Nyamukapa, Robertson, Mushore, Takaruza, & Gregson, Submitted for publication), which suggests that more transient households may also be excluded from the PWR procedure.

However, it should also be noted that many households were also missing data from the community verification exercise and there was little evidence of a systematic bias in the exclusion of households from this process. This suggests that there may have been a more general problem with the application of the community-based targeting methods, perhaps resulting from poor training or the imposition of geographical community boundaries used in the community-randomized trial, which may have included households that were unfamiliar to the community leaders involved in the PWR and verification exercises.

There was better agreement between the census-based information about household socio-demographic characteristics and the information from the community verification exercise. This was to be expected as the community representatives were asked to validate the information collected in the census rather than to provide information independently of the census. The tendency to over-report chronically-ill, disabled, paternally orphaned, and double orphaned household members in the census, relative to the community verification, may have occurred because households were aware that the survey was linked to the cash transfer program and may have over-stated the frequency of these household members in the census in order to benefit from the program. This suggests that the community verification exercise was effective at reducing inclusion errors relative to the census-based method alone. However, it is also possible that households hide illness, disability, and orphanhood for fear of stigma and community representatives may not always be aware of the status of these household members.

The asset-based wealth index and the PWR method showed moderate success at targeting vulnerable children: both methods reached children who were more likely to be suffering from poor educational and social outcomes. The asset-based wealth index method was more effective at targeting children with poor outcomes than the PWR procedure. However, both methods failed to target a large proportion of children with poor outcomes and the efficiency of the methods was generally low—few children with poor outcomes were reached per child targeted. The asset-based wealth index method was more efficient than the PWR procedure at reaching children with poor outcomes and the efficiency did not decline as a greater proportion of the poorest households (i.e., 28% compared with 18%) were targeted.

We found that census-based wealth indices were the most effective and efficient way of targeting children with poor outcomes. However, all five of the methods investigated were relatively inefficient and failed to reach a large proportion of vulnerable children. In terms of effectiveness and efficiency, combining the census-based wealth index method with the PWR procedure offered few improvements over the asset-based wealth index alone.

It may be that household-based targeting using either quantitative or community-defined measures of wealth or poverty is not a particularly successful way of reaching children with poor outcomes. Alternative methods could involve directly targeting children with poor outcomes or perhaps reaching children through alternative channels e.g., in school or through community outreach work. Similarly, it may be that targeting the poorest children excludes other types of vulnerable children e.g., orphans or those caring for sick adults. Previous work using the Manicaland Cash Transfer Trial census data found that asset-based wealth index was more effective and efficient at targeting children with poor outcomes compared with targeting based on household-level socio-demographic characteristics such as caring for orphaned, chronically ill, or disabled household members (Robertson *et al*., 2012).

The advantages of the PWR procedure were highlighted by our qualitative study: the method allows community involvement in the selection process and thereby increases community ownership and acceptance of poverty alleviation programs. The increased transparency and perceived fairness of the method can reduce the potential for conflict within communities, especially in a context where the majority of households are struggling to meet their basic needs. Previous studies have found that the involvement of communities in decisions about the distribution of local resources to improve child wellbeing can strengthen community responses to the needs of vulnerable children (Skovdal *et al*., 2013).

PWR is also cheap and can be carried out relatively quickly, although our method for carrying out the census—asking members to convene at a central meeting point within the community was much quicker than traditional methods where each household is visited by a research assistant (Gregson *et al*., 2006). Thus the PWR method may be more cost-effective, despite being somewhat less effective and efficient than the census at identifying the poorest households. Comparing the cost-effectiveness of different types of targeting method is an important area for future work.

In light of the positive community response to their involvement in the targeting process, and the program more generally (Skovdal *et al*., 2013, in press), and the possible benefits in terms of reducing inclusion errors, it is clear that providing community members with opportunities to participate in poverty alleviation programs have the potential to open up for possibilities that have implications for the success and sustainability of cash transfer programs. Despite the small number of in-depth interviews and focus group discussions that were conducted, the added value of these possibilities needs to be recognized and should be explored further in future programs adopting community-based targeting methods. This argument resonates with the growing interest and recognition of the community response to HIV (Campbell *et al*., 2013; Gregson *et al*., 2013; Rodriguez-García *et al*., 2013).

Nonetheless, it is also clear that both the community-based and census-based methods had serious difficulty reaching vulnerable children, with the asset-based wealth index offering some advantages in terms of effectiveness and efficiency over the PWR procedure. Given the frequency of the use of socio-demographic and wealth data, derived from household surveys and community committees, to target cash transfer programs (Ministry of Community and Social Services (MCDSS) & German Technology Cooperation (GTZ), 2007; Robertson *et al*., 2013; Schubert & Huijbregts, 2006) and the increasing popularity of wealth ranking procedures (Pronyk *et al*., 2006), our results are of some concern. Further work is required to improve methods for targeting social welfare interventions to the poorest households and the most vulnerable children.

## Figures and Tables

**Table 1:** Summary of study informants

	Individual interviews		Focus groups		Total no. of interviews
	Adults	Children	Adults	Children	
Key informants	15	0	1 (9 people)	0	16
Cash transfer beneficiaries	6	1	1 (9 people)	0	8
Conditional cash transfer beneficiaries	5	3	0	0	8
Non-beneficiaries	5	0	1 (9 people)	0	6
Total no. of people	31	4	27	0	38
					62

**Table 2:** Coding framework—Program ownership optimizing the impact of cash transfer initiatives

Codes	Basic themes	Organizing themes
Working with local structures	Community-based committees to take an active role in the implementation of the program	Locating a cash transfer program in a social context through community involvement
Community committee elected democratically		
Community committee involved in program implementation		

Participatory wealth ranking (PWR)	Community members involved in the selection of beneficiaries	
Benefits of PWR		
Community verification		
Selection done fairly		
Transparency		

Support of local leaders	Through community involvement, local resources are made available	Community involvement enhances ownership and success
Local knowledge		
Community embeddedness improves communication		
Community monitoring		
Ownership and appropriation of program		

Solidarity for beneficiaries	Community involvement can improve openness and dialog	
Recognition of how the community benefits from the program		
Selection done fairly		
Enhances community dialog and support		

There is a limit to community involvement	Challenges faced by community committee members	Obstacles and barriers to community involvement
Challenges faced by community committees		

**Table 3:** Comparison of households caring for children less than 18 years and children living in households with missing and non-missing community data

	PWR data			Community-based socio-demographic data		
	Not missing	Missing	*p*-Value	Not missing	Missing	*p*-Value
*Household level*						
% Poorest 20% of households	18.24%	16.62%	0.067	18.08%	15.57%	0.061
% Contains maternal orphan	24.93%	20.92%	<0.001	23.87%	25.36%	0.317
% Contains paternal orphan	41.12%	33.22%	<0.001	39.34%	38.61%	0.672
% Child headed	0.80%	0.74%	0.760	0.76%	1.01%	0.429
% Chronically ill member	35.19%	34.52%	0.560	34.81%	37.42%	0.117
% Disabled member	11.49%	9.64%	0.011	11.16%	10.03%	0.306
% Female headed	43.23%	36.44%	<0.001	41.98%	38.15%	0.032
% Elderly headed	15.92%	15.99%	0.933	15.95%	15.83%	0.932
% Labor constrained	27.48%	25.17%	0.024	26.75%	29.03%	0.140
Mean number of children	2.66	2.51	<0.001	2.63	2.56	0.112

*Child level*						
% Not fully vaccinated (0–4 years)	38.11%	27.67%	<0.001	35.87%	33.68%	0.334
% Without birth certificate (0–4 years)	53.75%	55.59%	0.214	53.84%	57.84%	0.075
% Attending school less than 80% of days (6–12 years)	20.16%	22.62%	0.008	20.38%	24.30%	0.005
% Attending school less than 80% of days (13–17 years)	27.88%	26.87%	0.414	27.89%	25.12%	0.137
% Female	50.07%	48.82%	0.085	49.83%	49.40%	0.697
Mean age	9.05	8.85	0.006	9.01	8.96	0.694

**Table 4:** Measurement of the agreement between census-based and community-based information on characteristics of household vulnerability among households caring for children less than 18 years

	Census data		Community data		kappa	*N*
	%	*N*	%	*N*		
Maternal orphan in household	24.00	10,481	24.54	9,635	0.57	9,588
Paternal orphan in household	39.28	10,240	37.24	9,637	0.62	9,362
Double orphan in household	18.47	10,196	17.41	9,635	0.56	9,322
Child-headed household	0.78	10,461	0.84	8,379	0.25	8,318
Chronically ill household member	35.03	10,522	28.84	9,637	0.48	9,624
Disabled household member	11.06	10,507	8.39	9,637	0.56	9,610

**Table 5:** Effectiveness, coverage, and efficiency of targeting methods based on an asset-based wealth index, participatory wealth ranking, and combinations of both methods with respect to reaching children with poor outcomes

	Birth not registered (0–4 years)			Not fully vaccinated (0–4 years)			Attending school less than 80% of days (6–12 years)			Attending school less than 80% of days (13–17 years)		
*(1) Effectiveness: Age- and sex-adjusted odds ratios comparing the likelihood of poor outcomes among targeted and non-targeted children*												
	AOR	95% CI	*N*	AOR	95% CI	*N*	AOR	95% CI	*N*	AOR	95% CI	*N*
Asset-based wealth index (poorest 18%)	1.68	1.47–1.90	6,217	1.02	0.89–1.17	5,697	1.51	1.35–1.68	11,098	1.89	1.67–2.15	7,766
Asset-based wealth index (poorest 28%)	1.76	1.58–1.97	6,219	1.01	0.90–1.14	5,699	1.55	1.29–1.87	11,358	1.96	1.70–2.25	7,931
Poorest category of PWR exercise	1.50	1.31–1.71	4,793	0.82	0.71–0.95	4,402	1.23	1.10–1.37	8,692	1.34	1.19–1.51	6,160
Combined (inclusive)	1.65	1.46–1.86	4,764	0.78	0.68–0.89	4,376	1.36	1.23–1.52	8,637	1.56	1.39–1.74	6,119
Combined (exclusive)	1.71	1.41–2.08	4,764	1.14	0.93–1.40	4,376	1.26	1.07–1.48	8,637	1.75	1.46–2.10	6,119

*(2) Coverage: Percentage of children with poor outcomes reached by each targeting method*												
	%	95% CI	*N*	%	95% CI	*N*	%	95% CI	*N*	%	95% CI	*N*
Asset-based wealth index (poorest 18%)	24.88	23.43–26.33	3,396	21.72	19.93–23.51%	2,049	24.23	22.48–25.98%	2,307	22.40	20.64–24.16%	2,152
Asset-based wealth index (poorest 28%)	39.26	37.62–40.90	3,398	34.50	32.44–36.56%	2,049	37.97	35.99–39.95%	2,307	34.90	32.89–36.91%	2,152
Poorest category of PWR exercise	29.80	28.04–31.56	2,597	23.49	21.47–25.51%	1,690	33.30	31.10–35.50%	1,757	34.96	32.71–37.21%	1,719
Combined (inclusive)	42.27	40.36–44.18	2,581	33.73	31.47–35.99%	1,684	44.65	42.32–46.98%	1,747	45.14	42.78–47.50%	1,708
Combined (exclusive)	12.82	11.53–14.11	2,581	11.22	9.71–12.73%	1,684	12.42	10.87–13.97%	1,747	12.88	11.29–14.47%	1,708

*(3) Efficiency: Number of children with poor outcomes reached per child targeted by targeting method*												
	#	95% CI	*N*	#	95% CI	*N*	#	95% CI	*N*	#	95% CI	*N*
Asset-based wealth index (poorest 18%)	0.64	0.61–0.66	1,324	0.36	0.33–0.39	1,233	0.27	0.25–0.29	2,094	0.39	0.36–0.42	1,229
Asset-based wealth index (poorest 28%)	0.63	0.61–0.65	2,114	0.36	0.34–0.38	1,962	0.26	0.25–0.28	3,359	0.37	0.35–0.39	2,024
Poorest category of PWR exercise	0.60	0.58–0.63	1,280	0.34	0.32–0.37	1,153	0.22	0.21–0.24	2,602	0.32	0.30–0.34	1,872
Combined (inclusive)	0.61	0.59–0.63	1,784	0.35	0.33–0.37	1,631	0.23	0.22–0.25	3,351	0.34	0.32–0.36	2,297
Combined (exclusive)	0.65	0.60–0.69	513	0.40	0.36–0.45	471	0.24	0.21–0.26	916	0.39	0.35–0.43	566

*(4) Percentage of all children reached by each targeting method*												
	0–4 years (%)	6–12 years (%)	13–17 years (%)									
Asset-based wealth index (poorest 18%)	21.08	18.91	15.99									
Asset-based wealth index (poorest 28%)	33.65	30.26	26.12									
Poorest category of PWR exercise	26.49	29.74	30.53									
Combined (inclusive)	37.11	38.65	37.77									
Combined (exclusive)	10.70	10.61	9.32									

## Footnotes

1 The formula used for the weighted *κ* statistic was $\kappa =1-\frac{\sum_{i=1}^{5}\sum_{j=1}^{5}|i-j|\cdot x_{\mathrm{ij}}}{\sum_{i=1}^{5}\sum_{j=1}^{5}|i-j|\cdot x_{i.}\cdot x_{.j}/n}$ where *x_ij_* is the number of households in wealth quintile *i* and PWR group *j*, $x_{i.}=\sum_{j=1}^{5}x_{\mathrm{ij}}$ and $x_{.j}=\sum_{i=1}^{5}x_{\mathrm{ij}}$.
